# Out-of-Hospital Cervical Ripening With a Synthetic Hygroscopic Cervical Dilator May Reduce Hospital Costs and Cesarean Sections in the United States—A Cost-Consequence Analysis

**DOI:** 10.3389/fpubh.2021.689115

**Published:** 2021-06-18

**Authors:** Sita J. Saunders, Rhodri Saunders, Tess Wong, Antonio F. Saad

**Affiliations:** ^1^Coreva Scientific, Königswinter, Germany; ^2^Medicem, Inc., Boston, MA, United States; ^3^Department of Obstetrics and Gynecology, University of Texas Medical Branch at Galveston, Galveston, TX, United States

**Keywords:** cervical ripening, mechanical dilator, prostaglandins, induction of labor, health economics, cost-consequence analysis, cesarean section, outpatient

## Abstract

**Objective:** Out-of-hospital (outpatient) cervical ripening prior to induction of labor (IOL) is discussed for its potential to decrease the burden on hospital resources. We assessed the cost and clinical outcomes of adopting an outpatient strategy with a synthetic hygroscopic cervical dilator, which is indicated for use in preinduction cervical ripening.

**Methods:** We developed a cost-consequence model from the hospital perspective with a time period from IOL to post-delivery discharge. A hypothetical cohort of women to undergo IOL at term with an unfavorable cervix (all risk levels) were assessed. As the standard of care (referred to as *IP-only*) all women were ripened as inpatients using the vaginal PGE2 insert or the single-balloon catheter. In the comparison (*OP-select*), 50.9% of low-risk women (41.4% of the study population) received outpatient cervical ripening using a synthetic hygroscopic cervical dilator and the remaining women were ripened as inpatients as in the standard of care. Model inputs were sourced from a structured literature review of peer-reviewed articles in PubMed. Testing of 2,000 feasible scenarios (probabilistic multivariate sensitivity analysis) ascertained the robustness of results. Outcomes are reported as the average over all women assessed, comparing OP-select to IP-only.

**Results:** Implementing OP-select resulted in hospital savings of US$689 per delivery, with women spending 1.48 h less time in the labor and delivery unit and 0.91 h less in the postpartum recovery unit. The cesarean-section rate was decreased by 3.78 percentage points (23.28% decreased to 19.50%). In sensitivity testing, hospital costs and cesarean-section rate were reduced in 91% of all instances.

**Conclusion:** Our model analysis projects that outpatient cervical ripening has the potential to reduce hospital costs, hospital stay, and the cesarean section rate. It may potentially allow for better infection-prevention control during the ongoing COVID-19 pandemic and to free up resources such that more women might be offered elective IOL at 39 weeks.

## Introduction

National data indicate an induction of labor (IOL) rate of over one in four deliveries in the United States (US) (1). Elective IOL at 39 weeks is expected to add to medically indicated IOL in light of recent evidence (2–4). Inducing low-risk women at 39 weeks was shown to reduce the risk of cesarean sections, hypertension during pregnancy, and neonatal respiratory morbidity in comparison to expectant management (2–4). Routine adoption of elective IOL at 39 weeks raises the concern of overburdening existing resources (5). Although Grobman et al. reported that it might not increase healthcare resource utilization as expected, providing evidence for decreases in antepartum hospitalization, visits, treatments, and tests; the women in the IOL group spent 6 h longer in the labor and delivery (L&D) unit (6). Implementing an out-of-hospital (outpatient) strategy for cervical ripening has the potential to shorten the time spent in the L&D unit and significantly decrease cesarean sections (7, 8).

In this manuscript, we performed a cost-consequence analysis and present how implementing outpatient cervical ripening with a synthetic hygroscopic cervical dilator may impact outcomes for low-risk women undergoing (elective) IOL at term and their babies, and healthcare providers. We compared a purely inpatient strategy (IP-only) against a proportion of eligible women being ripened mechanically in the outpatient setting(OP-select).

## Materials and Methods

We performed a cost-consequence analysis from an average US hospital perspective, focusing on the IOL care pathway: starting from hospital admission for IOL with an unfavorable cervix and ending at post-delivery discharge. In addition to costs, we report differences in clinical, cost-impacting outcomes between the IP-only and OP-select strategies. This publication was written following the *Consolidated Health Economic Evaluation Reporting Standards* (CHEERS) checklist which is provided in the Supplementary Material (9).

### Cervical Ripening Agents

This study considers the impact of adopting mechanical cervical ripening with an osmotic, synthetic hygroscopic cervical dilator (Dilapan-S^®^, MEDICEM Technology, Czechia) for outpatient cervical ripening. As the current standard of care (SOC), a vaginal PGE2 (Cervidil^®^, Ferring Production Inc, USA) insert is used unless contraindicated; in such cases, the intracervical single-balloon catheter (Foley) is used. In an alternative analysis, the vaginal PGE2 insert is replaced by the intracervical PGE2 gel (Prepidil^®^, Pfizer USA). Throughout the rest of this manuscript and in the Supplementary Material, we use the following names to refer to each ripening agent: *synthetic hygroscopic cervical dilator* for Dilapan-S^®^, *balloon catheter* for Foley, *PGE2 insert* for Cervidil^®^, and *PGE2 gel* for Prepidil^®^.

### Model Setup

**Figure 1:**
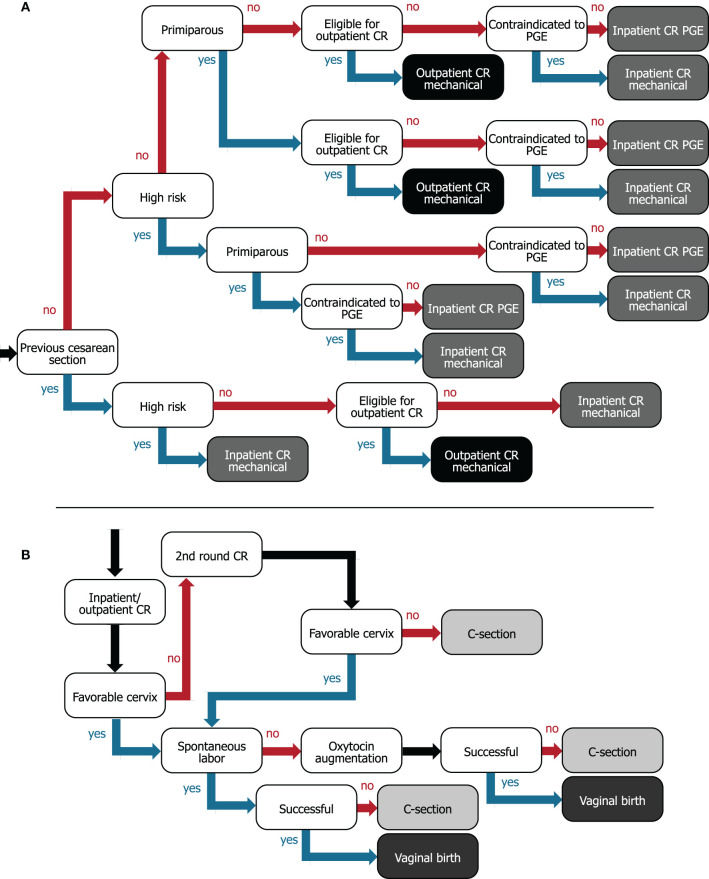
Decision trees for **(A)** flow of women from hospital admission to assigning women to inpatient or outpatient for preinduction cervical ripening and **(B)** cervical ripening/IOL to delivery care pathway. For **(A)**, internal nodes (white boxes) describe patient characteristics and leaves (gray/black boxes) represent the type of ripening agent administered and whether women are ripened in the inpatient (gray) or outpatient (black) setting. **(B)** The internal nodes (white boxes) represent events or interventions, and the leaves (gray boxes) represent vaginal (dark gray) or cesarean birth (light gray) as the two possible outcomes. CR, cervical ripening; C-section, cesarean section; PGE, prostaglandin.

Cost and clinical consequences were modeled for a cohort of women using a decision-tree model, programmed in Microsoft^®^ Excel^®^, that follows general guidance from International Society for Pharmacoeconomics and Outcomes Research (ISPOR) (10, 11). A cost-consequence model was selected to assess both the economic and clinical impact and it is commonly used to evaluate medical devices. It was determined that a decision-tree model appropriately describes the modeled care pathway (Figure 1). The time horizon was from admission to hospital for IOL to post-delivery discharge (~2–4 days); discounting of costs was not required because the time horizon was <1 year. Hospital costs and patient characteristics were estimated utilizing a representative US population. Input parameters are given in Tables 1, 2 and the Supplementary Material.

**Table 1:** Model inputs for patient characteristics, costs, and hospital stay.

**Model input**	**Base case [SD]**	**Data source**
High-risk deliveries	18.6% [1.86]	(2)
Previous C-section	12.3% [1.23]	(12)
Primiparous	31.4% [3.14]	(12)
Contraindicated to PGE2 insert/gel	21.0% [6.30]	Assumption from clinical practice of AS
**Purchase of ripening agent**		
PGE2 insert	$297.47 [29.75]	(13)
PGE2 gel	$365.17 [36.52]	(13)
Balloon catheter	$7.81 [0.78]	(14)
SHCD	$304.00 [30.40]	Medicem Inc. list price, 2020; mean 3.8 rods (15) at $80 each
Administration of ripening agent	$361.73 [36.75]	(16)
**Monitoring cost during ripening**		
PGE2 insert/gel	$250.00 [75.00]	Assumption
Mechanical ripening	$200.00 [60.00]	Assumed lower as no ECG monitoring required
Oxytocin augmentation	$176.03 [17.60]	(17)
Standard vaginal delivery	$12,875.14 [6688.38]	(18)
Standard cesarean delivery	$18,131.87 [9943.48]	(18)
Uterine rupture treatment	$21,558.74 [2155.87]	(19)
Cost for NICU stay after delivery	$33,694.54 [3369.45]	(20)
Serious perinatal morbidity cost	$3,634.08 [363.41]	(21)
Serious maternal morbidity cost	$4,988.22 [498.82]	(18)
L&D unit cost per hour	$133.46 [13.35]	(16)
**Time from IOL to delivery**		
PGE2 insert/gel	23.50 h [2.35]	(22)
Balloon catheter	22.79 h [2.28]	(23)
SHCD	25.29 h [2.53]	(24)
**Hospital stay in postpartum unit**		
After vaginal delivery	48 h [4.8]	(25, 26)
After cesarean delivery	72 h [7.2]	(26)

*IOL, induction of labor; L&D, labor and delivery; SD, standard deviation; NICU, neonatal intensive care unit; h, hour(s); balloon catheter, transcervical single-balloon catheter; SHCD, synthetic hygroscopic cervical dilator. All costs are inflated to 2020 USD*.

**Table 2:** Model inputs for clinical events.

**Model input**	**Incidence [SD]**	**RR [95% CI] or hours [min-max]**	**Data source**
**PGE2 insert vs. synthetic hygroscopic cervical dilator**			
Primary cesarean sections (primiparous)	25.5% [2.7]	0.668 [0.295–1.476]	(12); CRR (24, 27)
Primary cesarean sections (multiparous)	8.1% [1.7]	0.983 [0.325–2.919]	(12); CRR (24, 27)
VBAC	13.3% [2.1]	1.070 [0.710–1.620]	(28, 29)
Oxytocin augmentation	55.3% [3.1]	1.540 [1.350–1.760]*	Uses the balloon catheter as a proxy (27)
Failed 1st attempt cervical ripening	38.5% [6.4]	1.190 [0.504–2.868]	(27); CRR, cervix unfavorable after 24 h, (27) & 2nd round dilator HCD vs. balloon (24)
NICU admissions	7.4% [1.6]	0.820 [0.650–1.040]	Uses the balloon catheter as a proxy (27)
Uterine rupture	0.4% [0.4]	0.200 [0.010–4.120]	Uses the balloon catheter as a proxy (27)
Perinatal serious morbidity or death	2.0% [0.9]	0.480 [0.250–0.930]*	(27)
Maternal serious morbidity or death	0.3% [0.3]	0.200 [0.010–4.120]	Uses the balloon catheter as a proxy (27)
**Inpatient vs outpatient setting for preinduction cervical ripening**			
Cesarean sections	Not required	0.63 [0.46–0.86]*	(8)
L&D unit time saved	Not required	5.51 h [2.00–9.01]	(7)

RR, relative risk; CI, confidence interval; SD, standard deviation; VBAC, vaginal birth after cesarean section; *statistically significant outcome; CRR, combined relative risk; NICU, neonatal intensive care unit; L&D, labor and delivery. Studies compare inpatient with outpatient ripening using the balloon catheter, which is used as a proxy for the synthetic hygroscopic cervical dilator.

### Literature Search

Clinical evidence was identified using a methodical, structured search of PubMed. Search specifications are given in the Supplementary Material. All inputs for incidences of clinical outcomes and comparative relative risks were taken from the captured literature, favoring meta-analyses and randomized controlled trials. Studies were selected for the most recent or the most appropriate population. Manual searches of PubMed, Google Scholar, and the National Center for Health Statistics (USA) were used to identify US-specific population characteristics and healthcare costs if no suitable data were identified in literature already captured by the structured search.

### Population Characteristics

All women admitted for IOL for term deliveries (>37 weeks gestational age), classified with an unfavorable cervix requiring cervical ripening, were considered in this hypothetical cohort. Further characteristics considered in the model were: risk level of the pregnancy (low or high; high-risk subjects were not eligible for outpatient mechanical ripening), women with a previous cesarean section, parity (primiparous or multiparous), and contraindication to the prostaglandin used. Parameter values are listed in Table 1. Similarly to the ARRIVE trial (2), we defined a low-risk pregnancy as the absence of any condition considered to be a maternal or fetal indication for delivery before 40 weeks 5 days (e.g., hypertensive disorders of pregnancy or suspected fetal-growth restriction) (2). The 18.6% used to indicate high-risk pregnancies was taken from the trial exclusion criteria: out of 50,581 women, 7,560 had a maternal or obstetrical condition and 1,854 had a fetal or placental condition (2). We considered all other women to be of low risk and eligible for outpatient cervical ripening.

Women were assigned to a cervical-ripening method based on their baseline characteristics as demonstrated in the decision tree in Figure 1A. If a woman with a previous cesarean section is otherwise low risk, we assumed that she could be offered outpatient cervical ripening with the synthetic hygroscopic cervical dilator, since the risk of a uterine rupture is low and this event typically occurs during the active phase of labor (at which point women return to the hospital) (30). All women with a previous cesarean section were considered contraindicated to receive prostaglandins (31).

### Comparison of Cervical-Ripening Strategies

The model takes the hospital's viewpoint and compares cohorts of women who are treated as described in the following two strategies.

**IP-only**: SOC where all women are ripened in the inpatient setting. The vaginal PGE2 insert is used for cervical ripening unless contraindicated, in which case the balloon catheter is used.

**OP-select**: Women are eligible for outpatient ripening with the synthetic hygroscopic cervical dilator if they are low risk [81.4% given the ARRIVE trial (2)]. The number thereof, which are assigned to outpatient ripening in the model, is regulated by a parameter that can be set from 0 to 100 percent. The base case is 50.9%: midpoint of women considered in broad- (60.67%) and limited-use (41.15%) outpatient scenarios by Son et al. (16). This corresponds to 41.4% (81.4/100 × 50.9) of the entire cohort that underwent outpatient cervical ripening. Remaining low- and high-risk women (58.6% of the cohort) were assigned to SOC inpatient cervical ripening (as per IP-only).

Outpatient in the US context requires clarification, as it is a term used for outpatient hospital departments, the physician's office, birthing centers, or ambulatory surgery centers. The costs and resources associated with each option vary. Here, we consider outpatient cervical ripening to occur as follows: (1) The ripening agent is administered in the hospital, (2) the woman goes home for the ripening phase, (3) she returns to the hospital for either a second attempt at cervical ripening or for delivery (± oxytocin augmentation). The woman is given instructions by the hospital staff about when to return to the hospital.

### Clinical Pathway Modeled From IOL to Birth

The full IOL to delivery pathway is illustrated in Figure 1B. Women first receive a ripening agent, and after ripening, the cervical status is checked. If the first attempt at cervical ripening is not successful, a second round of cervical ripening is attempted using the same ripening agent. Although this might not always reflect current practice, we used the same ripening agent, because combinations of ripening agents would require clinical efficacy data for the exact combinations applied, for which sufficient data are not available. Women in outpatient ripening who require a second round return to the hospital for reinsertion of the mechanical dilator. We used an incidence of 18.6% (SD = 4.8) for the number of women undergoing a second ripening attempt that fail again and require a cesarean section, estimated by the number undelivered after 48 h divided by the number undelivered after 24 h from Blackwell et al. (32). If the cervix is favorable after ripening but labor does not occur spontaneously, oxytocin is administered to induce uterine contractions. Although American College of Obstetricians and Gynecologists (ACOG) guidelines specify that oxytocin should be administered after a failed second ripening attempt (33), we have omitted this step because (1) the same incidence of failed second attempts is applied in the model for both IP-only and OP-select strategies, and (2) data on the incidence of failed second attempts are scarce for all required ripening agents. Trial of labor ends in a vaginal or cesarean delivery. Operative vaginal deliveries are not considered.

### Clinical Outcomes for Ripening Agents

Incidences of studied outcomes were identified for the selected prostaglandin (the PGE2 insert or gel). Comparisons between the mechanical ripening agents (the synthetic hygroscopic cervical dilator or the balloon catheter) and the selected prostaglandin were recorded as relative risks. Outcomes directly related to the delivery were: cesarean-section rate (considering primary cesarean-section rates for primi- and multiparous women separately), vaginal births after previous cesarean (VBAC), requiring oxytocin augmentation, and the number of women failing the first ripening attempt. Adverse events considered were: NICU admissions, uterine rupture, serious perinatal morbidity or death, and serious maternal morbidity or death. Clinical events were selected according to the most recent Cochrane review on mechanical methods for IOL (27). Hospital stay is assessed as time in (1) the L&D unit and (2) the postpartum recovery unit. Table 2 lists model inputs to do with clinical events; further model inputs are given in the Supplementary Material. Postpartum stay was estimated by multiplying the rate of cesarean sections and vaginal births by the average length of postpartum stay for the respective birth category (Table 1) (25, 26). Comparisons depend on the ripening agent used and the inpatient/outpatient setting (for cesarean section and L&D time). Relative risks are multiplied for agent and setting comparisons. When clinical evidence is not directly available for the required input, we utilize combined relative risks of the closest comparisons for a reasonable estimate. For example, a combined relative risk (*CRR*) comparing the PGE2 insert (*P*) with the synthetic hygroscopic cervical dilator (*H*) using data from the balloon catheter (*B*) equals:

$$\begin{array}{l} CRR_{H}^{P}=\;EXP\left(ln\left(RR_{B}^{P}\right)+ln\left(RR_{H}^{B}\right)\right), \end{array}$$

where RR denotes a relative risk between the two agents indicated. We used outcomes for the balloon catheter or laminaria tent for the synthetic hygroscopic cervical dilator whenever specific outcomes are not available. The synthetic hygroscopic cervical dilator was shown to be non-inferior to the balloon catheter (24). Evidence on the double-balloon catheter is also used for the single-balloon catheter as these were combined in the recent Cochrane review (27). Inpatient vs. outpatient cervical-ripening studies so far report outcomes related to the balloon catheter and we selected meta-analyses covering the most studies (Table 2) (7, 8).

### Costs

Cost inputs are given in Table 1. Considered costs can be roughly separated into three categories: Costs relevant to (1) induction of labor, (2) type of delivery, and (3) adverse events. All costs extracted from references are inflated to 2020 USD. Total care-pathway costs are mean costs per delivery.

#### IOL

We considered purchase, inpatient monitoring, and administration costs for the cervical-ripening agent, cost of oxytocin augmentation, and the cost for time in the L&D unit.

#### Type of Delivery

We used an average US-hospital delivery cost for a standard vaginal or cesarean delivery (18). This cost includes hospital stay, resource utilization, and intervention costs. We assume the standard delivery cost to be representative of a delivery without induction of labor.

#### Adverse Events

We considered care costs for uterine rupture, NICU admissions, and serious maternal or perinatal morbidity. The categories of adverse events may overlap but due to a lack of combined safety data, we consider these independently.

### Model Calculations

Expected incidences of cesarean sections and vaginal births were calculated for the IP-only and OP-select strategies separately. This was done by multiplying the relevant incidences and relative risks (Tables 1, 2) along the branches of the decision trees depicted in Figure 1. For example, 2.3% of women in the model population had a previous cesarean section [12.3% taken from Hehir et al. (12)] and were considered to be high risk [18.6% calculated from Grobman et al. (2)] which was calculated as:

$$\begin{array}{l} 12.3\times 18.6÷100=2.3. \end{array}$$

Incidences for the PGE2 insert were used as the reference, which were then multiplied by the relative risk for each of the other cervical-ripening agents used and inpatient/outpatient setting. Resulting incidences of cesarean sections and vaginal births were finally multiplied by associated clinical events and costs and then combined. In this way, model results are an average outcome for the same cohort of hypothetical women undergoing cervical ripening with the IP-only vs. OP-select, and results can be scaled to any population size.

### Scenario Analyses

We performed three scenario analyses that differ from the model base case described above.

- Women with a previous cesarean (i.e., trial of labor after cesarean—TOLAC) but an otherwise uncomplicated pregnancy are ripened inpatient instead of outpatient.
- Only clinical outcomes that were significantly different were included. Others were set to be equal between ripening agents and settings.
- Only primiparous women are assessed.

### Sensitivity Analyses

The performed multivariate probabilistic sensitivity analysis explored the robustness of model outcomes by choosing randomly selected values within the specified range of uncertainty for all variables. For this, a seeded (i.e., reproducible) uniform random number was sampled for each parameter between 0.001 and 0.999 and is used as the point at which to sample the parameter's cumulative distribution function. Each parameter is assigned a distribution: lognormal for relative risks and a normal otherwise, described by a mean and standard deviation. To eliminate potential for “non-sense” values (e.g., a negative incidence) each parameter has a specified logical range. Unless given directly in or calculated from the source, we used a standard deviation of 10% by default and 30% when an assumption had to be made. For binary clinical events, we applied the binomial proportion confidence interval with a normal distribution to estimate uncertainty; the maximum population size used for this estimation was 1,000. The multivariate sensitivity analysis was repeated 2,000 times and outcomes were summarized using the 95% credible interval (CrI) and by the percentage of sampling runs that benefitted the OP-select over the IP-only strategy.

Because costs can be highly variable, we performed an additional univariate deterministic sensitivity analysis for all cost parameters for the scenario where the PGE2 insert was used for inpatients. For every cost parameter, the given standard deviation in Table 1 was used to convert the mean value into an upper- and lower-bound cost, which was entered into the model; all other parameters remained the same. Total expected cost savings were plotted as a tornado plot in the Supplementary Material.

## Results

We examined the potential cost and clinical consequences of providing cervical ripening in the outpatient setting. As described in the methods, analyses were performed on a computational model, developed to represent the cervical ripening and IOL care pathway in the US. Our analysis informs on the feasibility of implementing the OP-select in comparison to the IP-only strategy, considering the impact on hospital budgets, resources, and clinical outcomes. In IP-only, 79.0 and 21.0% of women were ripened as inpatients with the PGE insert and the balloon catheter, respectively. In OP-select, 46.3 and 12.3% were ripened as inpatients with the PGE2 insert and the balloon catheter, respectively; and 41.4% of women were ripened as outpatients with the synthetic hygroscopic cervical dilator. All findings, though methodologically robust, are estimates from a computational model and require supporting, real-world studies to be considered as high-level evidence of benefit.

### Cost Saving Potential

The OP-select strategy was estimated to save hospitals US$689 per delivery, Table 3. Cost savings consisted of US$199 for standard delivery costs, US$304 for IOL, and US$186 for treating adverse events. These average savings were achieved by assigning 50.9% of low-risk women to outpatient cervical ripening (equivalent to 41.4% of the entire population) and averaging over the entire cohort of women.

**Table 3:** Model cost and clinical outcomes in the base case comparing IP-only with OP-select strategies.

**Model output**	**IP-only vs. OP-select (with IP PGE2 insert)**		**IP-only vs. OP-select (with IP PGE2 gel)**	
Cost per delivery (total) Standard delivery Induction of labor Adverse events	–$689 –$199 –$304 –$186	($17,893 vs. $17,204) ($14,099 vs. $13,900) ($1,246 vs. $941) ($2,548 vs. $2,362)	–$866 –$196 –$350 –$320	($22,693 vs $21,826) ($14,101 vs. $13,904) ($1,321 vs. $972) ($7,271 vs. $6,950)^*^
Cesarean sections	−3.78	(23.28 vs. 19.50%)	−3.74	(23.32 vs. 19.58%)
VBACs (% of TOLACs)	9.11	(13.30 vs. 22.41%)	9.11	(13.30 vs. 22.41%)
NICU admissions	−0.44	(7.12 vs. 6.68%)	−0.84	(20.96 vs. 20.11%)
Uterine rupture	−0.10	(0.33 vs. 0.23%)	−0.10	(0.33 vs. 0.23%)
Perinatal SMD	−0.34	(1.78 vs. 1.44%)	−0.26	(3.43 vs. 3.17%)
Maternal SMD	−0.08	(0.25 vs. 0.17%)	−0.08	(0.25 vs. 0.17%)
Time in hospital (total)	−2.39 h	(76.94 vs. 72.31 h)	−2.38 h	(76.95 vs. 72.37 h)
Time in L&D	−1.48 h	(23.35 vs. 21.87 h)	−1.48 h	(23.35 vs. 21.87 h)
Postpartum recovery	−0.91 h	(53.59 vs. 52.68 h)	−0.90 h	(53.60 vs. 52.70 h)
Oxytocin augmentation	9.97	(60.85 vs. 70.81%)	1.82	(61.49 vs. 63.31%)

*TOLAC, trial of labor after previous cesarean; VBAC, vaginal birth after previous cesarean; SMD, serious morbidity or death; IP, inpatient; L&D, labor and delivery*.

* *High adverse events costs for the PGE2 gel are higher because there is a much greater uncertainty in the input for its NICU admissions (27). Differences are given in percentage points unless otherwise stated. Costs are given in 2020 USD*.

**Figure 2:**
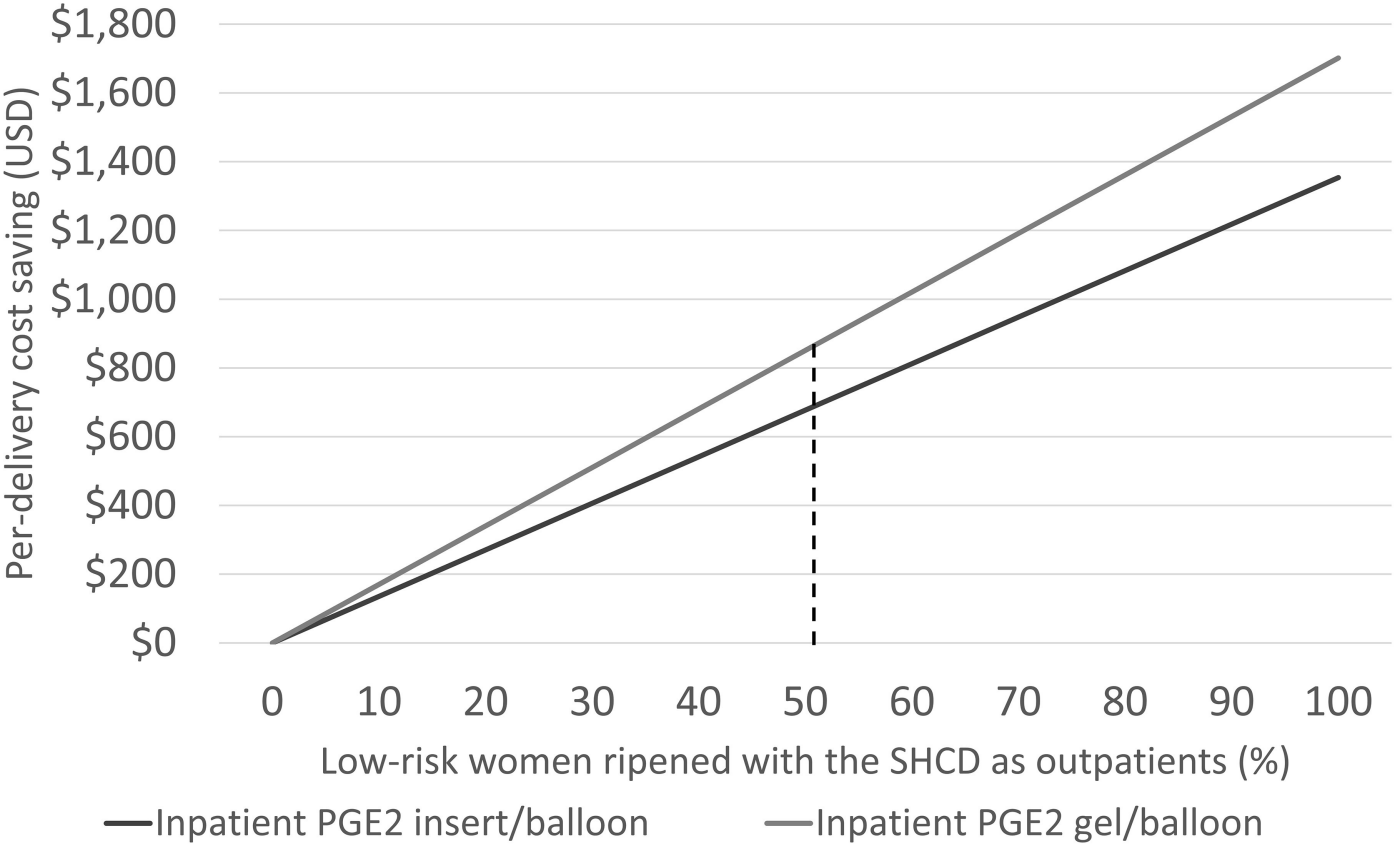
Illustrating the cost-saving potential by increasing the number of low-risk women for outpatient cervical ripening with the synthetic hygroscopic cervical dilator (SHCD) from 0 to 100%. The dashed line represents the model base case at 50.9%.

Considering only women switching from inpatient to outpatient ripening, the mean cost saving per outpatient delivery was US$1,663: US$480 for standard delivery, US$735 for IOL, and US$448 for treating adverse events. Cost savings linearly increased with increasing numbers of low-risk women assigned to outpatient ripening: from US$0 (no outpatients) to US$1,354 (*all* low-risk woman are ripened as outpatients) per delivery, Figure 2. Cost savings are presented in Table 4 for a variety of outpatient proportions alongside sensitivity analyses. It is noteworthy that the model predicts cost savings even for low numbers of outpatients. Presented results represent use of the PGE2 insert for inpatient cervical ripening of non-contraindicated women, results for use of PGE2 gel are also provided in Table 3 and Figure 2.

**Table 4:** Multivariate probabilistic sensitivity analyses for increasing percentages of low-risk women ripened out of hospital with the synthetic hygroscopic cervical dilator.

**% of LRW in outpatient setting**	**Cost difference**		**Cesarean sections**		**VBAC**	
	**Base case** **(median)**	**95% CrI**	**Base case** **(median)**	**95% CrI**	**Base case** **(median)**	**95% CrI**
50.9%^*^	–$689 (–$574)	–$1,798–$355	−3.8% points (−3.3)	−6.6–1.4% points	9.1% points (8.2)	−0.8–17.5% points
20%	–$271 (–$225)	–$706–$140	−1.5% points (−1.3)	−2.6–0.6% points	3.6% points (3.2)	−0.3–6.9% points
40%	–$542 (–$451)	–$1,413–$279	−3.0% points (−2.6)	−5.2–1.1% points	7.2 % points (6.4)	−0.7–13.8% points
60%	–$812 (–$676)	–$2,119–$419	−4.5 % points (−3.9)	−7.6–1.7% points	10.7% points (9.6)	−1.0–20.6% points
80%	–$1,083 (–$901)	–$2,826–$558	−6.0% points (−5.2)	−10.3–2.2%-points	14.3% points (12.9)	−1.3–27.5% points
100%	–$1,354 (–$1,127)	–$3,532–$698	−7.4% points (−6.5)	−12.9–2.8% points	17.9% points (16.1)	−1.7–34.4% points

*LRW, low-risk women; CrI, credible interval; VBAC, vaginal birth after cesarean section;*

* *model base case setting. Results are given only for the inpatient use of the PGE2 insert instead of the PGE2 gel. Costs are given in 2020 USD*.

### Less Time Spent in the Hospital

The presented model estimates that in the base case, time saved in hospital will be 1.48 h in the L&D unit and 0.91 h in the postpartum recovery unit: a total hospital stay of −2.39 h, when averaged over the entire cohort (Table 3). For outpatient women ripened with the synthetic hygroscopic cervical dilator, 3.57 h less time is predicted for the L&D unit and 2.16 h less in the postpartum recovery unit (−5.73 h in total) in comparison to inpatient SOC ripening. Note that in the OP-select strategy, the increased time from induction to labor required for the synthetic hygroscopic cervical dilator in comparison to the PGE insert or the balloon catheter (Table 1) was added to the in-hospital L&D time and not to the time in outpatient ripening. In practice one may expect more time saved in the L&D unit for women in the outpatient setting.

### Outpatient Mechanical Cervical Ripening May Reduce Cesarean Births and Lead to Minor Decreases in Adverse Events

Cesarean sections were decreased by 3.78 percentage points (23.2 vs. 19.5%), Table 3. For women undergoing TOLAC, we observed a substantial 9.11 percentage-point increase in VBACs (13.3 vs. 22.4%). Minor decreases in serious adverse events—NICU admissions, uterine ruptures, and perinatal and maternal severe morbidity and death—were also estimated by the model (Table 3). Results were comparable when the PGE2 gel was used for inpatients (Table 3).

### Scenario Analyses

**Figure 3:**
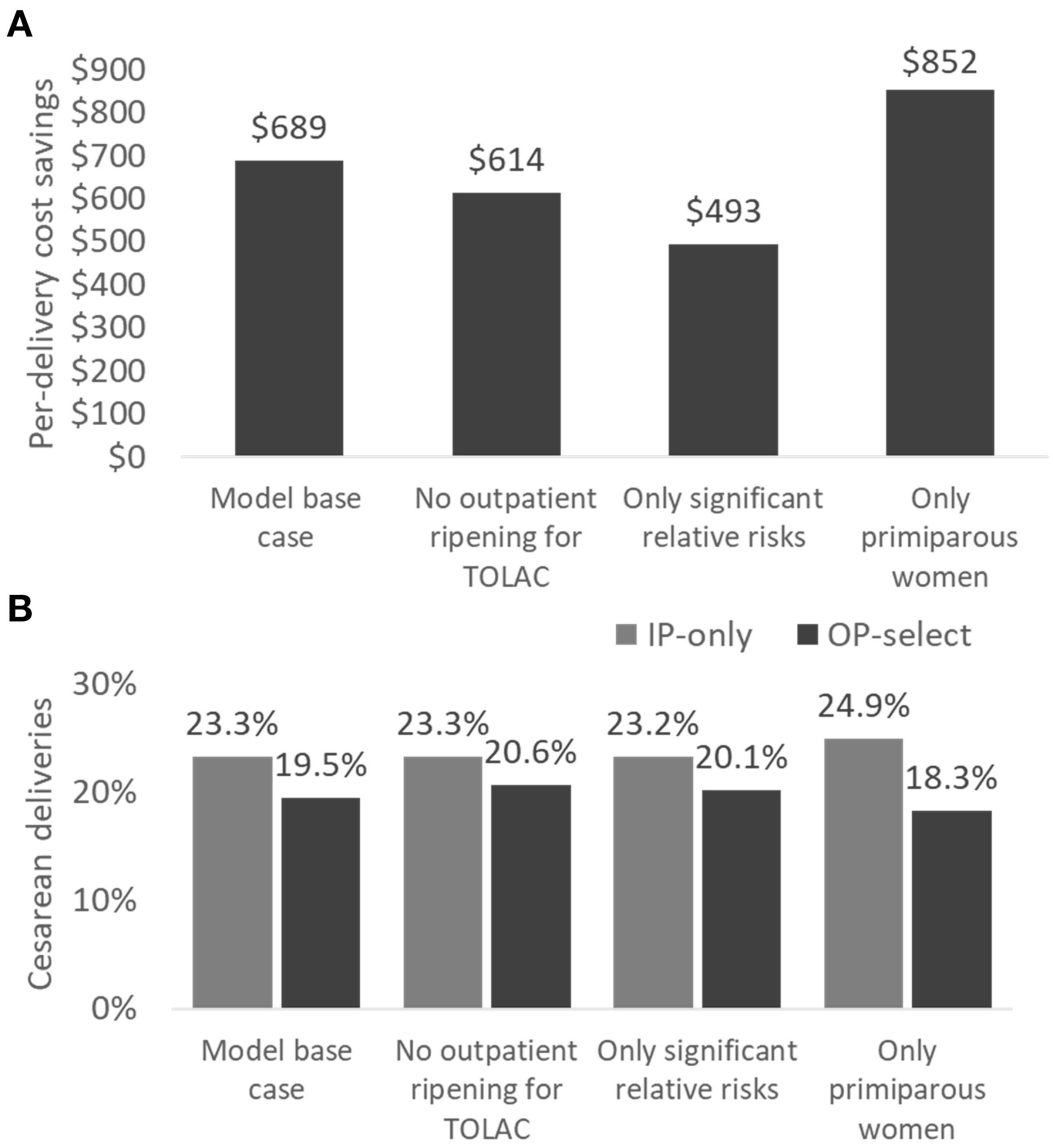
Scenario analyses comparing per-delivery cost savings **(A)** and cesarean sections **(B)** in IP-only vs. OP-select strategies. Scenario analyses: (1) model base case, (2) women for TOLAC are ripened in the hospital only, (3) all non-significant relative risks for clinical events are set to 1.0, and (4) only primiparous women are assessed. TOLAC, trial of labor after cesarean section.

For each scenario analysis (Figure 3), cost savings ranged between US$493 and US$852 per delivery with the highest savings expected for a primiparous population. Retaining women for TOLAC in the hospital did not lead to a substantial difference in results, decreasing savings by US$75 per delivery. Cesarean sections were decreased in each scenario analysis with the greatest change predicted for primiparous women (24.9 vs. 18.3%). The recent Cochrane review also supports a greater benefit of mechanical ripening agents for primiparous in comparison to multiparous women (27). In addition, primiparous women often require longer time for cervical ripening and thus present the best opportunity for time savings from outpatient ripening (34). On the flipside, however, these first-time mothers might be more anxious than veteran mothers, and they may need additional information and support if they are to feel comfortable at home.

### Sensitivity Analyses

After exploring uncertainty in a multivariate probabilistic sensitivity analysis, including combinations that are unlikely to occur in real clinical settings, the model predicts cost savings in 90.5%, decreased cesarean sections in 90.5%, and increased VBAC births in 95.5% of all input-parameter settings. In a substantial proportion of possible input settings, our model estimates both a cost and a clinical benefit when adopting the OP-select in comparison to the IP-only strategy (Table 4). Multivariate sensitivity analyses show robust benefits even when only a low number of women are assigned to outpatient ripening.

In the univariate sensitivity analysis of cost parameters (Supplementary Material), the cost for cesarean and vaginal deliveries had the greatest impact on cost savings. However, it is to be expected that the overall cost ratio between cesarean vs. vaginal deliveries is more similar than fluctuations in absolute costs across the US. Differences in the cost ratio is what mostly affects overall cost savings. All other cost parameters did not impact outcomes substantially. Notably, none of the univariate analyses resulted in a cost increase for the OP-select vs. the IP-only scenario.

## Discussion

Few FDA-indicated cervical ripening methods are available with a safety profile suitable for use outside of the hospital setting. It has been suggested that mechanical methods may be most suited to facilitate outpatient cervical ripening (30, 35, 36). In comparison to the balloon catheter, Saad et al. found that the synthetic hygroscopic cervical dilator allowed for a statistically significant increase in the number of women able to perform their daily activities, and their ability to get some relaxation time and sleep (24). After insertion, the single-balloon catheter protrudes from the introitus and is usually kept under tension, while the synthetic hygroscopic cervical dilator remains mostly in the cervical canal, allowing for more freedom of movement (24). These factors make returning home a more attractive option when using the synthetic hygroscopic cervical dilator. Mechanical ripening was reported to be less effective than prostaglandins at achieving delivery within 24 h (27) however, using the outpatient strategy provides the woman more time for cervical ripening while spending less time in the L&D room.

Cost savings were US$689 per delivery with reductions in cesarean deliveries of 3.78%-points, and 2.39 h less time in the hospital. With the ongoing COVID-19 pandemic, there is an additional incentive to reduce the burden on hospital resources—and to safely keep patients out of the hospital. Furthermore, publishing on outpatient cervical ripening increased dramatically in 2020 showing a global increase in interest (7, 8, 16, 35–39). Figure 2 illustrates what one may expect in cost savings even for a very limited adoption of outpatient ripening. The time saved in the L&D unit alone could counteract the incremental expense for the synthetic hygroscopic cervical dilator or unexpected visits (16). Our model estimates suggest that hospitals can likely trial outpatient ripening with a very conservative set of women without expecting an increase in care costs—extending outpatient practice at a rate that is aligned with evidence from their local implementation.

Where comparisons are available, our results are generally aligned with other studies of mechanical cervical ripening and cervical ripening in the outpatient setting. In a costing study conducted in Australia, inpatient PGE2 gel was compared to outpatient Foley ripening (40). Mean costs per woman were not significantly different, however, the outpatient balloon-catheter group experienced fewer pre-delivery inpatient hours resulting in an incremental cost per patient hour prevented of AU$57 (40). For the USA, a cost-minimization and threshold analysis comparing the cost of inpatient vs. outpatient cervical ripening with a balloon catheter was published in 2020 (16). Here, Son and colleagues reported that in most plausible scenarios, outpatient ripening is cost saving: US$228.40/patient with broad use and US$73.48/patient with limited use. According to their model, outpatient ripening is no longer cost saving if time saved on L&D were <3.5 h, insertion visit cost >US$714, or facility cost/hour on L&D <US$61 (16). This study differs from the economic analysis presented here in the following aspects: (1) It compares only the balloon catheter used for both inpatient and outpatient, and (2) only the difference in ripening protocols was modeled with the assumption that there is no difference in adverse events or type of delivery. Here, we compared ripening agents commonly used as the SOC and included the full range of delivery care from admission to post-delivery discharge, including possible adverse events and changes in cesarean-section rates. Taken together these items provided a higher estimated cost difference—so although the magnitude varies, results agree that even limited use of outpatient ripening could reduce costs.

The potential for outpatient mechanical ripening to reduce cesarean sections adds to the reduction shown for elective IOL at 39 weeks, helping national initiatives to decrease cesarean-section rates in the US. Although the synthetic hygroscopic cervical dilator has a higher upfront purchasing cost than some alternatives and may lead to longer times from induction to delivery (22, 24), implementing the outpatient strategy may mitigate these factors. In addition, the synthetic hygroscopic cervical dilator is FDA cleared and has been shown to be non-inferior to other ripening agents with the same safety profile as the balloon catheter (non-FDA cleared) and with better patient satisfaction (24), and could thus be suited to the outpatient setting.

Health-economic models of patient cohorts are limited by the fact that they are based on average outcomes for an entire population and represent a simplification of real-life healthcare provision by design. For example, we model a second cervical-ripening attempt at a rate estimated by results presented in the literature (32), and we do not model any combinations of ripening agents. The model includes clinical inputs that present non-significant differences between the ripening agents being compared, and, if direct comparisons were missing, data was extrapolated to the closest comparator product. Although individual clinical outcomes did not differ significantly, in seven of 10 outcomes (Table 2), mechanical ripening and the outpatient setting were reported as having a relative risk less than one. Sensitivity and scenario analyses performed indicate that the base-case result was robust to uncertainty in all parameters. In a scenario analysis, when only considering significant clinical outcomes, cost savings and reductions in cesarean births were maintained.

It is typical that health-economic assessments are designed for specific countries and payer perspectives. The presented model uses population characteristics and costs representative of the US from the perspective of an average hospital. Therefore, presented results should be extrapolated with care to other countries with differing healthcare systems—and costs may need to be adjusted when the hospital in question differs considerably from the ones reported in this work.

## Conclusion

Adopting outpatient cervical ripening for low-risk women is predicted to reduce average delivery costs even when only a small proportion of women are ripened by this route. Our results suggest that hospitals can start with a limited number of women ripened in the outpatient setting and later expand their offer as evidenced by results from their own clinical practice without increasing overall costs. Further studies are required to confirm or contest these findings, but early evidence suggests that hospitals could start implementing outpatient cervical ripening while maintaining maternal-fetal safety parameters and potentially reducing cesarean deliveries. Outpatient ripening likely reduces the time women spend in hospital for a delivery by several hours, which aside from being cost saving, may be beneficial for infection prevention during the ongoing COVID-19 pandemic and afterwards may allow for elective IOL at 39 weeks to be offered to more women.

## Data Availability Statement

The original contributions presented in the study are included in the article/Supplementary Material, further inquiries can be directed to the corresponding author/s.

## Author Contributions

SS and RS designed and implemented the cost-consequences model. SS wrote the manuscript and performed the structured literature searches, revised by RS and TW. AS confirmed clinical processes, model inputs for the US practice, and interpreted model outcomes for obstetrics professionals. All authors contributed to the conceptual design of this work, revised the model and manuscript, and approved the final submission.

## Conflict of Interest

SS was an employee and RS was the owner of Coreva Scientific GmbH & Co KG, which received consultancy fees for performing, analyzing, and communicating the work presented here. TW was an employee of Medicem Inc., the US agent and initial importer of Dilapan-S^®^ and company funding this research. AS was an expert consultant and part of the advisory board for the sponsor. This study was funded by Medicem Inc. (USA). Medicem, Inc. contracted Coreva Scientific to perform this work and provided background details for the conceptual design of the analysis and reviewed the model and manuscript.
