# Supplementary material for: Out-of-Hospital Cervical Ripening With a Synthetic Hygroscopic Cervical Dilator May Reduce Hospital Costs and Cesarean Sections in the United States—A Cost-Consequence Analysis
Source: Front Public Health. 2021 Jun 18;9:689115. doi: 10.3389/fpubh.2021.689115 (PMC8249762; doi:10.3389/fpubh.2021.689115)
Supplement: Supplementary file 1 [file Data_Sheet_1.pdf]

Supplementary Material: Out-of-hospital cervical ripening with a synthetic hygroscopic cervical dilator may reduce hospital costs and cesarean sections in the United States—a cost-consequence analysis.

Sita J. Saunders, Rhodri Saunders, Tess Wong, Antonio F. Saad

1 Structured search details

Structured, reproducible searches were carried out using PubMed. All literature indexed by PubMed before the January 13th, 2021 and after January 1st, 2010, were captured by the presented search. The target of the search was to identify articles on:

1. Any economic models, systematic reviews, meta-analyses, and randomized controlled trials (RCTs) that were published within the previous decade on cervical ripening (Table 1-1). The focus of this search is on capturing major trends and results from the previous decade and to identify any previous work on economic assessments to give insight into the model design, for planning analyses, and for writing the manuscript.

2. Clinical studies, published within the previous decade, that mention at least one of Dilapan-S®, Foley or Cook® balloons, which are common mechanical agents for cervical dilation in use today (Table 1-2). The focus here is to capture all studies published on any one of the mechanical cervical ripening agents such that none are missed. For model inputs, we required direct comparisons with either the synthetic hygroscopic cervical dilator (Dilapan-S®), or the intracervical balloon Catheters (Cook® or Foley). Therefore, studies only have to mention either one of these mechanical methods and if they compare with prostaglandins, these are captured by default and do not need to be mentioned separately. Results reporting about simultaneous or consecutive use of ripening agents were not used as model inputs but were checked for contextual information.

Table 1-3 separates the articles from (1) and (2) into studies on the products of interest and others that do not mention these products. Here, we also see the total number of articles captured for separate categories.

Table 1-1. Structured searches in PubMed to identify major trends in cervical ripening

Index	Aim	Search string	Hits
**1**	Cervical ripening (incl. outpatient induction of labor)	"Cervical Ripening/analysis"[Majr] OR "Cervical Ripening/drug effects"[Majr] OR ((cervix[tiab] OR cervical[tiab]) AND (ripen[tiab] OR ripening[tiab] OR ripenings[tiab] OR maturing[tiab] OR softening[tiab] OR softenings[tiab] OR priming[tiab] OR preparation[tiab])) OR ((labor[tiab] OR labour[tiab]) AND (induced[tiab] OR induction[tiab] OR induce[tiab]) AND (outpatient[tiab] OR office[tiab] OR ambulant[tiab] OR "at home"[tiab]))	5,201
**2**	Induction of labor within the term period	(("Labor, Induced"[Majr] OR "induction of labor"[ti] OR "induction of labour"[ti] OR "labor induction"[ti] OR "labour induction"[ti] OR "inducing labor"[ti] OR "inducing labour"[ti]) AND (caesarean[tiab] OR cesarean[tiab])) NOT (preterm[tiab] OR pre-term[tiab] OR "early term"[tiab] OR postterm[tiab] OR post-term[tiab] OR premature[tiab])	1,708
**3**	TOLAC/VBAC	(("Trial of Labor"[Majr] AND (caesarean[tiab] OR cesarean[tiab])) OR (("trial of labor"[ti] OR "trial of labour"[ti]) AND (caesarean[tiab] OR cesarean[tiab])) OR tolac[tiab]) OR vbac[tiab] OR "vaginal birth after cesarean"[tiab] NOT vertebrobasilar[tiab]	1,647
**4**	High-level clinical evidence (systematic reviews, meta-analyses, RCTs)	"Controlled Clinical Trial" [Publication Type] OR "Meta-Analysis" [Publication Type] OR "Systematic Review" [Publication Type] OR "Randomized Controlled Trial" [Publication Type] OR ((randomized[tiab] OR randomised[tiab] OR prospective[tiab]) AND (controlled[tiab] OR controled[tiab] OR control[tiab] OR comparative[tiab] OR comparison[tiab]) AND (trial[tiab] OR study[tiab] OR studies[tiab] OR trials[tiab] OR analysis[tiab])) OR RCT[tiab] OR registries[tiab] OR registry[tiab] OR (systematic[tiab] AND review[tiab]) OR meta-analysis[tiab] OR metaanalysis[tiab]	1,288,074
**5**	Studies publishing costs or economic analyses	"Costs and Cost Analysis"[Mesh] OR "Cost-Benefit Analysis"[Mesh] OR "Cost of Illness"[Mesh] OR "Health Care Costs"[Mesh] OR "Cost Savings"[Mesh] OR "Direct Service Costs"[Mesh] OR "Hospital Costs"[Mesh] OR "Drug Costs"[Mesh] OR "Health Expenditures"[Mesh] OR "Health Resources/economics"[Mesh] OR "Economics, Hospital"[Mesh] OR "Economics, Medical"[Mesh] OR "Economics, Pharmaceutical"[Mesh] OR "budget-impact"[tiab] OR "budget impact"[tiab] OR "financial-impact"[tiab] OR "financial impact"[tiab] OR "cost-impact"[tiab] OR "cost-benefit"[tiab] OR "cost benefit"[tiab] OR "cost-utility"[tiab] OR "cost utility"[tiab] OR "cost-effective"[tiab] OR "cost effective"[tiab] OR "cost-effectiveness"[tiab] OR "cost effectiveness"[tiab] OR "health economic"[tiab] OR "health economics"[tiab] OR "health-economic"[tiab] OR ((USD[tiab] OR dollar[tiab] OR CAD[tiab] OR dollars[tiab]) AND (cost[tiab] OR costs[tiab] OR price[tw] OR expense[tiab] OR burden[tiab] OR economic[tiab] OR economics[tiab] OR financial[tiab]))	377,104

Index	Aim	Search string	Hits
**6**	Exclude off-topic applications	"cervical incompetence"[tiab] OR "cervical insufficiency"[tiab] OR "abortion"[tiab] OR "Abortion, Induced"[Majr] OR "termination of pregnancy"[tiab] OR "pregnancy termination"[tiab] OR "intrauterine foetal death"[tiab] OR "intrauterine fetal death"[tiab] OR IUFD[tiab] OR preterm[tiab] OR hysteroscopy[tiab] OR hysteroscopic[tiab] OR hysteroscopies[tiab] OR cancer[tiab] OR chemotherapy[tiab] OR disease[tiab] OR infertility[tiab] OR "cervical disc"[tiab] OR "cervical cages"[tiab] OR "cervical third"[tiab] OR "Models, Animal"[Mesh] OR horses[tiab] OR sheep[tiab] OR mouse[tiab] OR mice[tiab] OR "mechanical fatigue"[tiab] or elastography[tiab] OR tissue-mimicking[tiab] OR root[tiab] OR tooth[tiab] OR teeth[tiab] OR enamel[tiab] OR detine[tiab] OR dental[tiab] OR caries[tiab] OR contraception[tiab] OR contraceptive[tiab] OR contraceptives[tiab] OR lymph[tiab] OR lymphatic[tiab] OR simulation[tiab] OR simulated[tiab] OR simulators[tiab]	7,291,763
**7**	Exclude non-USA countries	(Japan[tiab] OR Japanese[tiab] OR Italy[tiab] OR Italian[tiab] OR China[tiab] OR Chinese[tiab] OR Germany[tiab] OR German[tiab] OR Netherlands[tiab] OR Dutch[tiab] OR Korea[tiab] OR Korean[tiab] OR Switzerland[tiab] OR Spain[tiab] OR Spanish[tiab] OR France[tiab] OR French[tiab] OR India[tiab] OR Indian[tiab] OR Canada[tiab] OR Canadian[tiab] OR Kingdom[tiab] OR UK[tiab] OR Ireland[tiab] OR Wales[tiab] OR Welsh[tiab] OR Irish[tiab] OR Brazil[tiab] OR Brazilian[tiab] OR Mexico[tiab] OR Mexican[tiab] OR Australia[tiab] OR Australian[tiab] OR Zealand[tiab] OR Pacific[tiab] OR European[tiab] OR Europe[tiab] OR Kenya[tiab] OR Kenya[tiab] OR Oman[tiab] OR Beijing[tiab] OR Nigeria[tiab] OR Taiwan[tiab] OR Emirates[tiab] OR Iceland) NOT (USA[tiab] OR American[tiab] OR America[tiab] OR "United States"[tiab])	2,080,860
**8**	Studies of interest	((#1 OR #2 OR #3) AND (#4 OR #5)) NOT #6 NOT #7	1,195
**9**	**Total studies of interest within the last decade**	**#8 AND 2010/01:2021/01/13[dp]**	**478**

Table 1-2. Structured searched in PubMed to identify clinical evidence for mechanical cervical ripening studies

Index	Aim	Search string	Hits
**10**	Products	dilasoft*[tiab] OR dilapan*[tiab] OR (osmotic[tiab] AND (dilator[tiab] OR dilators[tiab] OR dilation[tiab])) OR ((foley[tiab] OR cook[tiab]) AND (balloon[tiab] OR balloons[tiab] OR catheter[tiab] OR catheters[tiab])) OR ((balloon[tiab] OR balloons[tiab]) AND (catheters[tiab] OR catheter[tiab] OR dilator[tiab] OR dilators[tiab] OR induction[tiab] OR cervical[tiab] OR cervix[tiab])) OR single-balloon[tiab] OR double-balloon[tiab] OR single-balloons[tiab] OR double-balloons[tiab] OR ((mechanical[tiab] OR mechanic[tiab]) AND (cervix[tiab] OR cervical[tiab] OR labour[tiab] OR labor[tiab] OR dilation[tiab] OR ripening[tiab])) NOT (sweeping[ti] OR sweep[ti])	27,062
**11**	Exclude case reports	"Case Reports"[Publication Type] OR "case report"[tiab] OR "case study"[tiab]	2,267, 055
**12**	Studies of interest	((#1 OR #2 OR #3) AND #10) NOT (#11 OR #6)	486

Index	Aim	Search string	Hits
**13**	**Total studies of interest within the last decade**	**#12 AND 2010/01:2021/01/13[dp]**	**317**

Table 1-3. Summary of articles separated by mechanical cervical ripening agents and other categories

Index	Aim	Search string	Hits
**14**	Clinical studies for Dilapan-S, Foley and Cook	(#9 AND #10) OR #13	319
**15**	Other high-level evidence on TOLAC/VBAC only	(#9 AND #3) NOT (#1 OR #2 OR #14)	82
**16**	Other high-level evidence on induction of labor only	(#9 AND #2) NOT (#1 OR #3 OR #14)	123
**17**	Other high-level evidence on cervical ripening	#9 NOT (#14 OR #15 OR #16)	147
**18**	Total studies	#14 OR #15 OR #16 OR #17	671

Resulting literature was screened using Rayyan,[1] an online platform to support systematic reviews. The articles were screened against pre-selected criteria. Below we present the screening process for the product studies from which model input were sourced. Starting from 319 articles, 291 remained after screening (Figure 1). Included articles were later tagged by subtopics or relevance.

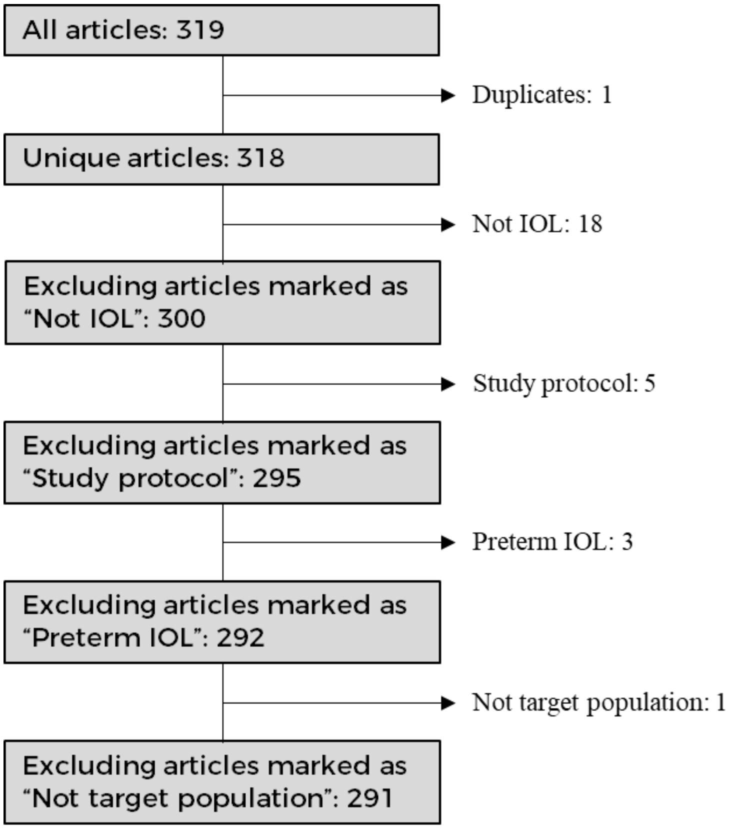

Figure 1. Screening comparative product studies for cervical ripening.

2 Remaining model inputs: comparative clinical events

The following tables list model inputs for clinical outcomes that are not specified in the main manuscript.

Table 4. Model inputs for relative risks for PGE2 insert vs. balloon

Model input	RR [95% CI]	Data sources
**Primary cesarean sections (primiparous)**	0.89 [0.59–1.33]	de Vaan 2019[2]
**Primary cesarean sections (multiparous)**	1.31 [0.65–2.63]	de Vaan 2019[2]

**VBAC**	1.00 [0.80–1.24]	Korb 2020[3]
**Oxytocin augmentation**	1.54 [1.35–1.76]*	de Vaan 2019[2]
**Failed 1st attempt CR**	0.96 [0.70–1.34]	cervix unfavorable after 24h, de Vaan 2019[2]
**NICU admissions**	0.82 [0.65–1.04]	de Vaan 2019[2]
**Uterine rupture**	0.20 [0.01–4.12]	de Vaan 2019[2]
**Perinatal SMD**	0.48 [0.25–0.93]*	de Vaan 2019[2]
**Maternal SMD**	0.20 [0.01–4.12]	de Vaan 2019[2]

RR—relative risk; CI—confidence interval; VBAC—vaginal birth after cesarean section; CR—cervical ripening; SMD—serious morbidity or death; *—statistically significant outcome; NICU—neonatal intensive care unit.

Table 5. Model inputs for clinical events for PGE2 gel vs. balloon and vs. synthetic hygroscopic cervical dilator

Model input	Incidence [SD]	PGE2 gel vs. balloon RR [95% CI]	PGE2 gel vs. HCD RR [95% CI]	Data sources
**Primary cesarean sections (primiparous)**	25.5% [2.7]	1.30 [0.86–1.95]	0.98 [0.43–2.15]	Hehir 2018[4]; de Vaan[2]; CRR, de Vaan 2019[2] & Saad 2019[5]
**Primary cesarean sections (multiparous)**	8.1% [1.7]	0.66 [0.16–2.78]	0.50 [0.08–3.09]	Hehir 2018[4]; de Vaan[2]; CRR, de Vaan 2019[2] & Saad 2019[5]
**VBAC**	13.3% [2.1]	1.00 [0.80–1.24]	1.07 [0.71–1.62]	Osterman 2020[6]; Korb 2020[3]; Maier 2018[7]
**Oxytocin augmentation**	61.0% [4.8]	1.08 [0.93–1.26]	1.08 [0.93–1.26]	de Vaan 2019[2]; uses balloon as a proxy
**Failed 1st attempt cervical ripening**	38.5% [6.4]	0.96 [0.70–1.34]	1.19 [0.50–2.87]	de Vaan 2019[2]; CRR, cervix unfavorable after 24h, de Vaan 2019[2] & 2nd round dilator HCD vs balloon, Saad 2019[5]
**NICU admissions**	2.2% [4.0]	0.88 [0.60–1.31]	0.88 [0.60–1.31]	de Vaan 2019[2]; uses balloon as a proxy
**Uterine rupture**	0.4% [0.4]	0.20 [0.01–4.12]	0.20 [0.01–4.12]	de Vaan 2019[2]; uses balloon as a proxy
**Perinatal serious morbidity or death**	3.6% [1.6]	0.78 [0.29–2.05]	0.78 [0.29–2.05]	de Vaan 2019[2]; uses balloon as a proxy
**Maternal serious morbidity or death**	0.3% [0.3]	0.20 [0.01–4.120]	0.20 [0.01–4.120]	de Vaan 2019[2]; uses balloon as a proxy

RR—relative risk; CI—confidence interval; SD—standard deviation; VBAC—vaginal birth after cesarean section; NICU—neonatal intensive care unit; no outcomes reach statistical significance; CRR—combined relative risk. When data was not available specifically for PGE2 gel, then data for the PGE2 insert, or a combination of PGE2, was taken as a proxy.

3 Univariate deterministic sensitivity analysis

For the scenario where the PGE2 insert was used in the inpatient setting, we performed a univariate deterministic sensitivity analysis for all cost parameters. Here the mean cost was converted to a lower and upper bound, given the standard deviation and resulting total cost savings were plotted and shown in Figure 2.

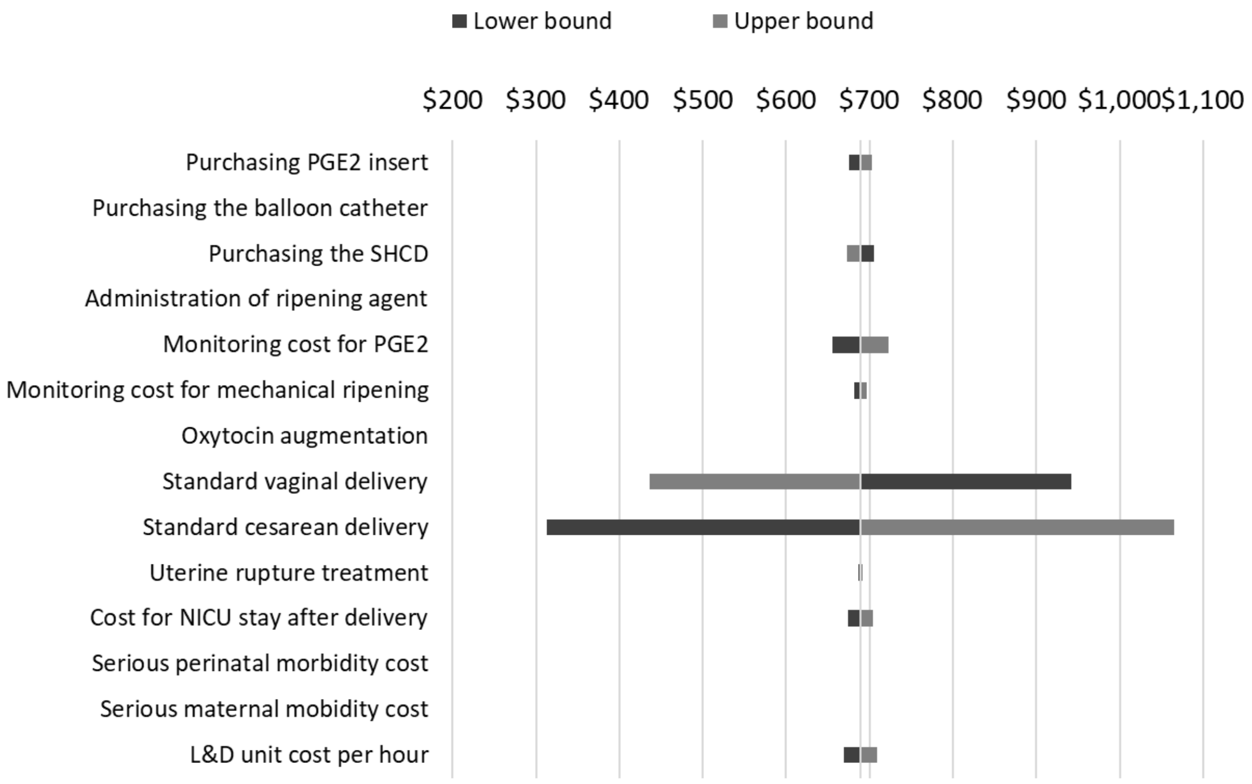

Figure 2. Univariate deterministic sensitivity analysis of cost parameters.[1] Total expected cost savings are given on the x-axis. PGE2 insert—vaginal dinoprostone inster; SCHD—synthetic hygroscopic cervical dilator; NICU—neonatal intensive care unit; L&D—labor and delivery. All costs are given in 2020 US$. The central line reflects the expected cost saving in the base case, US$689.

[1] Only cesarean and vaginal deliveries had a reported standard deviation in the source reference that is much greater than the 10% standard deviation used by default (see Table 1 in the manuscript for all standard deviations used). The monitoring costs had a 30% standard deviation because for these parameters assumptions were made.

4 CHEERS Checklist: reporting standards for health economic evaluations

Table 6 provides the Consolidated Health Economic Evaluation Reporting Standards (CHEERS) checklist[8] with references to page and line numbers in the original manuscript.

Table 6. CHEERS checklist: reporting standards for health economic evaluations

Selection/Item	Item	Recommendation	Page /Line*
Title and abstract			
Title	1	Identify the study as an economic evaluation or use more specific terms such as ‘‘cost-effectiveness analysis’’, and describe the interventions compared	1/0
Abstract	2	Provide a structured summary of objectives, perspective, setting, methods (including study design and inputs), results (including base case and uncertainty analyses), and conclusions	1-2 / 15-37
Introduction			
Background and objectives	3	Provide an explicit statement of the broader context for the study, Present the study question and its relevance for health policy or practice decisions	2-3 / 39-54
Methods			
Target population and subgroups	4	Describe characteristics of the base case population and subgroups analyzed, including why they were chosen	5/91-102 & 9-10/201-206
Setting and location	5	State relevant aspects of the system(s) in which the decision(s) need(s) to be made	3/56-59 & 4/73-

			81
Study perspective	6	Describe the perspective of the study and relate this to the costs being evaluated	3/56
Comparators	7	Describe the interventions or strategies being compared and state why they were chosen	3-4/63-71 & 5-6/1010-127
Time horizon	8	State the time horizon(s) over which costs and consequences are being evaluated and say why appropriate	4 / 78
Discount rate	9	Report the choice of discount rate(s) used for costs and outcomes and say why appropriate	4 / 79
Choice of health outcomes	10	Describe what outcomes were used as the measure(s) of benefit in the evaluation and their relevance for the type of analysis performed	7 / 146-172
Measurement of effectiveness	11a	Single study-based estimates: Describe fully the design features of the single effectiveness study and why the single study was a sufficient source of clinical effectiveness data	N/A
	11b	Synthesis-based estimates: Describe fully the methods used for identification of included studies and synthesis of clinical effectiveness data	4/ 83-89 & 7 / 146-172
Measurement and valuation of preference based outcomes	12	If applicable, describe the population and methods used to elicit preferences for outcomes	5/91-108
Estimating resources and costs	13a	Single study-based economic evaluation: Describe approaches used to estimate resource use associated with the alternative interventions. Describe primary or secondary research methods for valuing each resource item in terms of its unit cost. Describe any adjustments made to approximate to opportunity costs	N/A
	13b	Model-based economic evaluation: Describe approaches and data sources used to estimate resource use associated with	4/ 83-89 & 8-

		model health states. Describe primary or secondary research methods for valuing each resource item in terms of its unit cost. Describe any adjustments made to approximate to opportunity costs	9/174-185
Currency, price date, and conversion	14	Report the dates of the estimated resource quantities and unit costs. Describe methods for adjusting estimated unit costs to the year of reported costs if necessary. Describe methods for converting costs into a common currency base and the exchange rate	8 / 176
Choice of model	15	Describe and give reasons for the specific type of decision-analytical model used. Providing a figure to show model structure is strongly recommended	4/73-81
Assumptions	16	Describe all structural or other assumptions underpinning the decision-analytical model	3/52-54 & methods
Analytical methods	17	Describe all analytical methods supporting the evaluation. This could include methods for dealing with skewed, missing, or censored data; extrapolation methods; methods for pooling data; approaches to validate or make adjustments (such as half cycle corrections) to a model; and methods for handling population heterogeneity and uncertainty	7-10/ 146-225
Results			
Study parameters	18	Report the values, ranges, references, and, if used, probability distributions for all parameters. Report reasons or sources for distributions used to represent uncertainty where appropriate. Providing a table to show the input values is strongly recommended	3-10 / 56-225
Incremental costs and outcomes	19	For each intervention, report mean values for the main categories of estimated costs and outcomes of interest, as well as mean differences between the comparator groups. If applicable, report incremental cost-effectiveness ratios	11-12/238-268
Characterising uncertainty	20a	Single study-based economic evaluation: Describe the effects of sampling uncertainty for the estimated incremental cost and incremental effectiveness parameters, together with the impact of methodological assumptions (such as discount rate, study	N/A

		perspective)	
	20b	Model-based economic evaluation: Describe the effects on the results of uncertainty for all input parameters, and uncertainty related to the structure of the model and assumptions	13/281-293
Characterising heterogeneity	21	If applicable, report differences in costs, outcomes, or cost-effectiveness that can be explained by variations between subgroups of patients with different baseline characteristics or other observed variability in effects that are not reducible by more information	N/A
Discussion			
Study findings, limitations, generalisability, and current knowledge	22	Summarise key study findings and describe how they support the conclusions reached. Discuss limitations and the generalisability of the findings and how the findings fit with current knowledge	14-17/295-369
Other			
Source of funding	23	Describe how the study was funded and the role of the funder in the identification, design, conduct, and reporting of the analysis. Describe other non-monetary sources of support	18/401-403
Conflicts of interest	24	Describe any potential for conflict of interest of study contributors in accordance with journal policy. In the absence of a journal policy, we recommend authors comply with International Committee of Medical Journal Editors recommendations	18/390-393

Please note that the page and line references refer to the originally submitted document and may correspond to the final journal-formatted article.

5 Bibliography

1. Ouzzani M, Hammady H, Fedorowicz Z, Elmagarmid A. Rayyan-a web and mobile app for systematic reviews. *Syst Rev*. 2016;5(1):1-10. doi:10.1186/s13643-016-0384-4

2. de Vaan MD, ten Eikelder ML, Jozwiak M, et al. Mechanical methods for induction of labour. *Cochrane Database Syst Rev*. 2019;10(10):CD001233. doi:10.1002/14651858.CD001233.pub3

3. Korb D, Renard S, Morin C, Merviel P, Sibony O. Double-balloon catheter versus prostaglandin for cervical ripening to induce labor after previous cesarean delivery. *Arch Gynecol Obstet*. 2020;301(4):931-940. doi:10.1007/s00404-020-05473-x

4. Hehir MP, Ananth C V., Siddiq Z, Flood K, Friedman AM, D'Alton ME. Cesarean delivery in the United States 2005 through 2014: a population-based analysis using the Robson Ten Group Classification System. *Am J Obstet Gynecol*. 2018;219(1):105.e1-105.e11. doi:10.1016/j.ajog.2018.04.012

5. Saad AF, Villarreal J, Eid J, et al. A randomized controlled trial of Dilapan-S vs Foley balloon for preinduction cervical ripening (DILAFOL trial). *Am J Obstet Gynecol*. 2019;220(3):275.e1-275.e9. doi:10.1016/j.ajog.2019.01.008

6. Osterman MJK. Recent Trends in Vaginal Birth After Cesarean Delivery: United States, 2016-2018. *NCHS Data Brief*. 2020;(359):1-8.

7. Maier JT, Metz M, Watermann N, et al. Induction of labor in patients with an unfavorable cervix after a cesarean using an osmotic dilator versus vaginal prostaglandin. *J Perinat Med*. 2018;46(3):299-307. doi:10.1515/jpm-2017-0029

8. Husereau D, Drummond M, Petrou S, et al. Consolidated Health Economic Evaluation Reporting Standards (CHEERS)—Explanation and Elaboration: A Report of the ISPOR Health Economic Evaluation Publication Guidelines Good Reporting Practices Task Force. *Value Heal*. 2013;16(2):231-250. doi:10.1016/j.jval.2013.02.002
